# Perception thresholds and qualitative perceptions for electrocutaneous stimulation

**DOI:** 10.1038/s41598-022-10708-9

**Published:** 2022-05-05

**Authors:** Eva-Maria Dölker, Stephan Lau, Maria Anne Bernhard, Jens Haueisen

**Affiliations:** 1 Institute of Biomedical Engineering and Informatics, Technische Universität Ilmenau, Ilmenau, 98693 Germany; 2 Australian Institute for Machine Learning (AIML), School of Computer Science, The University of Adelaide, Adelaide, 5005 Australia

**Keywords:** Biomedical engineering, Health care

## Abstract

Our long-term goal is the development of a wearable warning system that uses electrocutaneous stimulation. To find appropriate stimulation parameters and electrode configurations, we investigate perception amplitude thresholds and qualitative perceptions of electrocutaneous stimulation for varying pulse widths, electrode sizes, and electrode positions. The upper right arm was stimulated in 81 healthy volunteers with biphasic rectangular current pulses varying between 20 and $2000\,\upmu \hbox {s}$. We determined perception, attention, and intolerance thresholds and the corresponding qualitative perceptions for 8 electrode pairs distributed around the upper arm. For a pulse width of $150\,\upmu \hbox {s}$, we find median values of 3.5, 6.9, and 13.8 mA for perception, attention, and intolerance thresholds, respectively. All thresholds decrease with increasing pulse width. Lateral electrode positions have higher intolerance thresholds than medial electrode positions, but perception and attention threshold are not significantly different across electrode positions. Electrode size between $15 \times 15\,\hbox {mm}^{2}$ and $40\times 40\,\hbox {mm}^{2}$ has no significant influence on the thresholds. Knocking is the prevailing perception for perception and attention thresholds while mostly muscle twitching, pinching, and stinging are reported at the intolerance threshold. Biphasic stimulation pulse widths between $150\,\upmu \hbox {s}$ and $250\,\upmu \hbox {s}$ are suitable for electric warning wearables. Within the given practical limits at the upper arm, electrode size, inter-electrode distance, and electrode position are flexible parameters of electric warning wearables. Our investigations provide the basis for electric warning wearables.

## Introduction

In the field of occupational safety, the warning of workers in hazardous situations is an important task that must be performed reliably. Warning signals should ideally allow for the communication of different meanings and the urgency of the situation to the user. For example, a railway track worker must be informed about an approaching train reliably^1^. Further, the information of which track it travels on and at what distance may need to be conveyed. Current methods use acoustic^1,2^ or visual^3^ warning signals. These warnings can fail under loud and/or low-visibility work situations. Therefore, we aim to develop a warning system that uses electrocutaneous stimulation through textile electrodes, which the user wears directly on the body. This novel system would allow transferring different information through amplitude, frequency, or spatio-temporal coding of the warning signal.

For our electrical warning system, we envisioned the following functional requirements: (1) as part of the personal safety equipment, the electrical warning signal must be perceivable, distinguishable from other stimuli, and unique; (2) the warning system should be able to transfer a small number of signal types; (3) the electrodes of the electrical warning system should be placed at a part of the body where: the wearing comfort is high, the electrodes are easy to attach, the sweat amount is low, the restriction of motion is small, the conflict potential with working and personal safety equipment is low, and the risk of muscle contraction and unexpected involuntary responses is minor. Additionally to the functional requirements, the electrical warning system should follow the norm ISO EN 60601^4^ that defines safety requirements and ergonomic requirements for medical electrical devices and systems as there is currently no norm for electrical warning systems. A third major requirement for our electrical warning system is the usability. The electrical warning system should be tolerated well by a user, wearable up to 8 h, light weight, integrated into the work clothes, and have stable electronics and energy supply.

Electrocutaneous stimulation for information transfer is already successfully applied in medical prostheses, giving the user sensory feedback^5^. A warning system, however, will use different stimulation amplitudes requiring a baseline study to find appropriate parameters. In a previous pilot study with four participants (f = 2, m = 2)^6^, we tested a broad range of stimulus and electrode parameters. We observed various types of qualitative perceptions (e.g. knocking, stinging, or itching) and 3 qualitative steps in the perception when stimulus amplitudes increased. These steps led us to the definition of three thresholds: (1) a just noticeable stimulus defines the perception threshold, (2) a stimulus drawing attention to itself defines the attention threshold, and (3) a stimulus generating intolerable perceptions defines the intolerance threshold. Moreover, we observed that the volunteers perceived the location of the stimulus partly at single electrode positions and partly between and beyond electrode positions. Consequently, in this study, we aim to investigate these thresholds and the qualitative and spatial perceptions of electrocutaneous stimulation in a larger study group comprising 81 volunteers. Additionally, we investigate the influence of pulse width, electrode size, and position.

## Methods

### Study group

The descriptive statistics of the study group are listed in Table 1. The ethics committee of the Faculty of Medicine of the Friedrich-Schiller-University Jena, Germany, approved the study. All methods were carried out in accordance with relevant guidelines and regulations. All participants gave written informed consent.

**Table 1:** Descriptive statistics of the study group.

Property	Quantity
Participants	$n=81$
Gender	Female: 29, male: 52
Age	27 years ± 7.8 years (mean ± standard deviation) youngest: 20 years, oldest: 52 years
Handedness	Right-handed: 72, left-handed: 9
Arm circumference (right arm)	30.3 ± 4.4 cm (mean ± standard deviation)

For the day of the experiment and the day before the experiment, participants were asked to get a sufficient amount of sleep, to not consume caffeine, nicotine, or alcohol, to drink enough (approx. 2 l), to do no hard, physical work or sports, and not treat the upper arms with skin cream.

### Experimental setup

**Figure 1:**
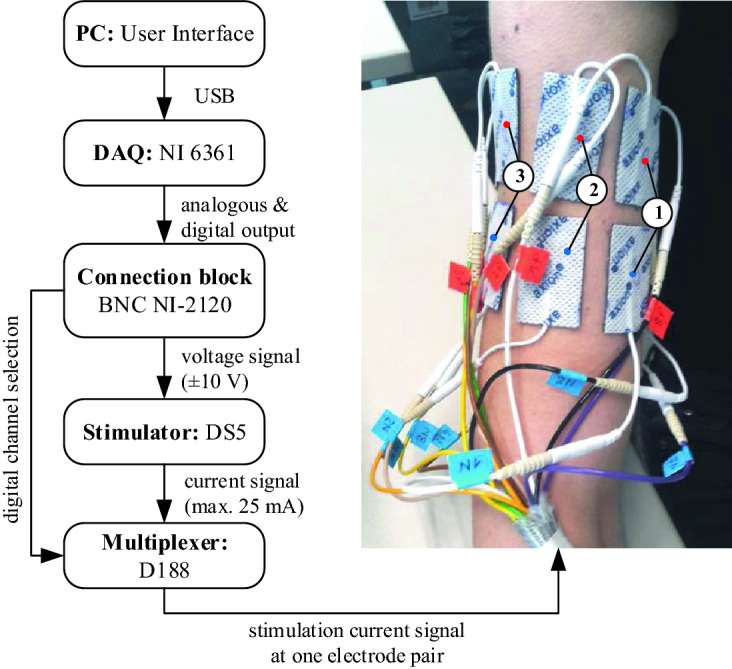
Schematic of the experimental setup. Left: block diagram of the stimulation setup. Right: example of electrodes at the upper arm (electrode pairs 1–3 of size 25 mm × 40 mm are visible). See text for detailed explanation.

Figure 1 shows a schematic of the experimental setup. The operator sets the stimulation parameters in the user interface of the in-house implemented program written in LabVIEW 2017 (National Instruments, Austin, TX, USA). This information is transferred to the data acquisition device (DAQ) NI USB-6361 (National Instruments, Austin, TX, USA) via USB. The output signal of the DAQ is transmitted to the connection block BNC NI-2120 (National Instruments, Austin, TX, USA), which sends a voltage signal (that encodes the desired stimulation signal) to the isolated bipolar constant current stimulator DS5 (Digitimer Ltd, Letchworth Garden City, UK). The DS5 converts the voltage signal input into a current signal output using the ratio 10 V:25 mA. The digital channel selection information is sent to the multiplexer D188 (Digitimer Ltd, Letchworth Garden City, UK), which activates one of its 8 output channels and thereby delivers the stimulus to the desired electrode pair.

We have chosen the upper arm as the stimulation site in consultation with electrophysiologists and neurologists. This location offers high wearing comfort and electrodes can be attached easily. Future wearable solutions like a cuff will not press into the arm intensely, allow an even attachment without creases, and offer minimal displacement during movements. The upper arm shows a low sweat amount and a minor restriction of motion. Further, this position offers a minimal conflict potential with working and personal safety equipment. The upper arm offers a reduced risk of muscle contractions or unexpected involuntary responses in comparison to sensitive areas like the face or the hands. In comparison to the lower limbs, the upper arm offers a better discriminability. Moreover, work-related external stimuli might be less common at the upper arm.

The 16 electrodes (re-useable self-adhesive TENS electrodes, axion GmbH, Leonberg, Germany) were placed pair-wise along the centerline between the shoulder joint and the elbow (Fig. 1) of the right arm. In circumferential direction, the electrodes were placed in distances of 1/8 of the arm circumference. For each electrode pair, one electrode was placed 5 mm above the center-line and the other 5 mm below. The electrode pairs were numbered consecutively, where electrode pair 1 corresponded to the anterior, 3 to the lateral, 5 to the posterior, and 7 to the medial position of the arm (Fig. 1). Electrode pairs were arranged in a vertical configuration with a top and a bottom electrode, because this results in better tolerated stimulation than transverse arrangements^6^. Stimulation at different electrode pair positions showed varied perceptions in a previous study^6^. Therefore, eight electrode pairs were chosen with one pair at each of the anterior, lateral, posterior, and medial positions and the remaining four in between (anterior-lateral, etc.). This allowed for an easier interpretation of the results with respect to physiological properties at these positions of the arm. Moreover, there was enough space to fit eight electrode pairs of the chosen size within all of the arm circumferences.

**Figure 2:**
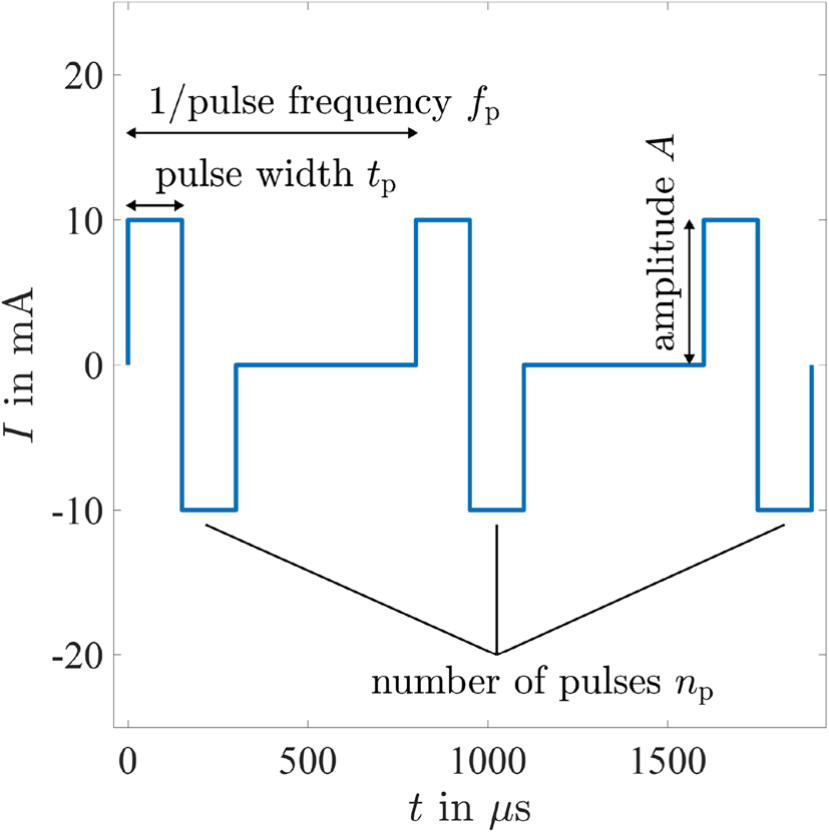
Example of the stimulation signal with biphasic rectangular pulses defined by amplitude ($A=10$ mA), pulse width ($t_{{\mathrm{p}}}=150\,\upmu \hbox {s}$ ), pulse frequency ($f_{{\mathrm{p}}}=1250$ Hz) and the number of pulses ($n_{{\mathrm{p}}}=3$).

The biphasic rectangular current stimulation signal (Fig. 2) was defined by the parameters: amplitude *A*, pulse width $t_{{\mathrm{p}}}$, pulse frequency $f_{{\mathrm{p}}}$ and number of pulses $n_{{\mathrm{p}}}$.

### Thresholds, qualitative and spatial perceptions

To find an operating range for an electrocutaneous warning system, three thresholds were defined in this study: (1) a just noticeable stimulus defines the perception threshold $A_{{\mathrm{p}}}$, (2) a stimulus drawing attention to itself defines the attention threshold $A_{{\mathrm{a}}}$, and (3) a stimulus generating intolerable perceptions defines the intolerance threshold $A_{{\mathrm{i}}}$.

In order to determine these thresholds, a single biphasic stimulation pulse (Fig. 2) of pulse width $t_{{\mathrm{p}}}= 150\,\upmu \hbox {s}$ was applied. The amplitude was increased gradually from 0 mA up to a maximum of 25 mA by steps of 0.1 mA, 0.2 mA, or 0.5 mA. The steps were chosen adaptively by the trained operator. Before the start of the experiment, the meaning of the three thresholds was explained to the participant. The operator always told the participant which threshold will be determined next such that the participant could focus on the threshold determination.

In this study, muscle twitches were considered to be an undesirable effect during fine-motor tasks. If muscle twitching occurred, the current threshold determination was stopped and the stimulation amplitude *A*, i.e. the motor threshold, was taken. This means that these three thresholds also incorporate motor threshold values that occurred before the threshold under determination was reached. The attention threshold acted as an indirect intensity measure for the perception of an attention-attracting stimulation.

At each threshold, the operator asked the participant about qualitative and spatial perception. For the qualitative perception, there were the following choices in the questionnaire: knocking, scratching, stinging, pain, muscle twitch, tickling, itching, pinching, squeezing. It should be noted that a painful perception is not necessarily identical to the intolerance threshold. Reaching the intolerance threshold means that the participant perceives the stimulus as intolerable, whereas the assigned qualitative perception is selected from the questionnaire and could be e.g. stinging, pinching, or also pain. A painful perception could also happen before reaching the intolerance threshold when the perception is painful but not intolerable. The categories of qualitative perception have been chosen according to our previous research^6^ and based on other fields of electrocutaneous stimulation^7–11^. The choices for the spatial perception included: between the electrodes, at both electrodes, at the upper or lower electrode, extending beyond electrodes, at other parts of the body. The above-mentioned definitions of the thresholds and the perception categories of the questionnaire were explained to the participant and queries from the participant were answered before starting the experiment.

### Experimental paradigm

The overall experiment took $138\pm 31$ min (mean ± standard deviation) including the preparation time and all sub-experiments. The experiment took place on the same day. The duration was mainly dependent on the participant. The mean durations  ±  standard deviations of each sub-experiment within the paradigm are indicated behind the corresponding sub-headers. The durations of the sub-experiments include the preparation times for the next sub-experiment (around 2–4 min). Note that the time for the sub-experiment ’Thresholds as functions of electrode size’ also contains the time of a short break (if necessary, around 10 min) and the attachment of the 8 electrode pairs. The time for these sub-tasks was not measured. Further, the last time point was noted at the beginning of the ’Reference threshold experiment’ at the end. Thus, approx. 2 min need to be added up to the total duration of the experiment and no mean value ± standard deviation is available for this final sub-experiment.

#### Preparation (30 ± 0 min)

The devices were switched on 30 min before the actual experiment for warming up. Additionally, the current stimulator DS5 (Fig. 1) was stabilized. For that purpose, a load resistor (1 k${\Omega }$) was connected to the DS5 and a series of stimulation pulses was sent through the load resistor multiple times. To optimize the transition impedance between the skin and the electrodes, the right upper arm of the participant was cleaned and moistened with a wet towel. The participant was asked to sit in a comfortable position, relax the arm, keep the view away from the experimental devices, and concentrate on the perception of the stimulation. During the experiment, the arm was positioned in a relaxed bent position on a table. The experiment was conducted at a room temperature of approx. $23\,^\circ \hbox {C}$.

In the following experimental parts, only electrode pair 3 (lateral position, Fig. 1, electrode size 25 × 40 mm) was attached to the arm of the participant and used for the experiment except for the investigation of the influence of electrode position.

#### Reference threshold experiment (14 ± 6 min)

During the reference threshold experiment, the threshold determination procedure was repeated at least 3 times until at least three consecutive series with similar thresholds were obtained. The similarity was evaluated by the trained operator. Using the repetitive threshold determination, the participant was able to learn the detection of the thresholds and the reporting of the perceptions. It ensured that the participant was used to the novel feeling of current stimulation. The mean values for perception, attention, and intolerance thresholds were calculated from the last 3 measurement series (denoted as repetition 1 in Fig. 4a). Regarding the qualitative and spatial perception, the most frequent out of the 3 measurement series were used. If three varying perceptions occurred the last one was selected.

#### Thresholds as a function of the pulse width $t_{{\mathrm{p}}}$ (18 ± 14 min)

Next, we conducted measurement series with varying pulse widths of $t_{{\mathrm{p}}}=$ 20, 50, 100, 150, 200, 250, 500, 1000, and 2000 μs. We determined the three thresholds including qualitative and spatial perceptions. The thresholds for $t_{{\mathrm{p}}}= 150\,\upmu \hbox {s}$ were also denoted as repetition 2 in Fig. 4a.

Next, another experiment (30 ± 10 min) took place, which is not reported in this paper.

#### Thresholds as a function of the electrode size (26 ± 15 min)

Another determination of the three thresholds was conducted with electrodes of sizes 40 mm × 40 mm, 25 mm × 40 mm, 20 mm × 20 mm, and 15 mm × 15 mm. Single pulses of pulse width $t_{{\mathrm{p}}}= 150\,\upmu \hbox {s}$ were used. The electrode pairs were placed at an edge-to-edge distance of 1 cm. Qualitative and spatial perceptions were documented. The threshold determination with electrode size 25 mm × 40 mm was denoted as repetition 3 in Fig. 4a.

Before the start of the next measurements, a short break was possible if desired by the participant. After the break, all 8 electrode pairs (electrode size 25 mm × 40 mm) were attached to the participant’s right arm.

#### Thresholds as a function of electrode position (20 ± 9 min)

For each of the 8 electrode pairs, the three thresholds and the corresponding qualitative and spatial perceptions were determined. The threshold determination for pair 3 is denoted as repetition 4 in Fig. 4a.

Next, another experiment (7 ± 4 min) took place, which is not reported in this paper.

#### Reference threshold experiment

For comparison, another threshold determination was conducted at the end of the experimental session using electrode pair 3 and single pulses of pulse width $t_{{\mathrm{p}}}= 150\,\upmu \hbox {s}$ (denoted as repetition 5 in Fig. 4a).

### Statistics and analysis

The experimental results were analyzed using MATLAB 2019 (The MathWorks, Inc., Natick, Massachusetts, USA). The results are visualized using Box–Whisker-plots and frequency distributions. Each Box–Whisker-plot contains a notch that visualizes the 95% confidence interval of the median of the corresponding distribution. If Box–Whisker-Plots are compared and their notches do not overlap, then it can be considered a statistically significant ($\alpha =0.05$) difference of the median values of both distributions^12,13^. These notches are used as a visual hypothesis test for the comparison of Box–Whisker-plots. The Lilliefors test^14^ ($\alpha _{\text {Bonferroni}}={0.05}/81\approx 0.001$) revealed that 25% of all measured distributions of thresholds were not normally distributed. Thus, the results are quantified by the median *M* and the interquartile range *IQR*.

For the analysis of the three thresholds in dependence of the investigated pulse widths, a non-linear decrease of the thresholds is expected with increasing pulse widths. To evaluate the decrease statistically, the pulse width $t_\text {p}$ was substituted by the assumed non-linear relation $x=1/t_\text {p}$. A linear regression $y=\beta _0+\beta _1\cdot x$ was calculated using the least squares method, where *y* represents the thresholds. The regression has been evaluated by the root mean square error (RMSE), the coefficient of determination $\hbox {R}^{2}$, and a t-test of the regression coefficients with^15^. To correct for the multiple testing, $\alpha$ was divided by the number of t-tests. In consequence, $\alpha =0.05/6=0.0083$ is used.

For the first reference threshold experiment, we presented absolute values of perception, attention, and intolerance thresholds. The values of all other experiments were normalized by the individual perception threshold determined from the reference threshold experiment for better comparability.

## Results

### Perception thresholds

#### Reference experiment and repetition of threshold determination

**Figure 3:**
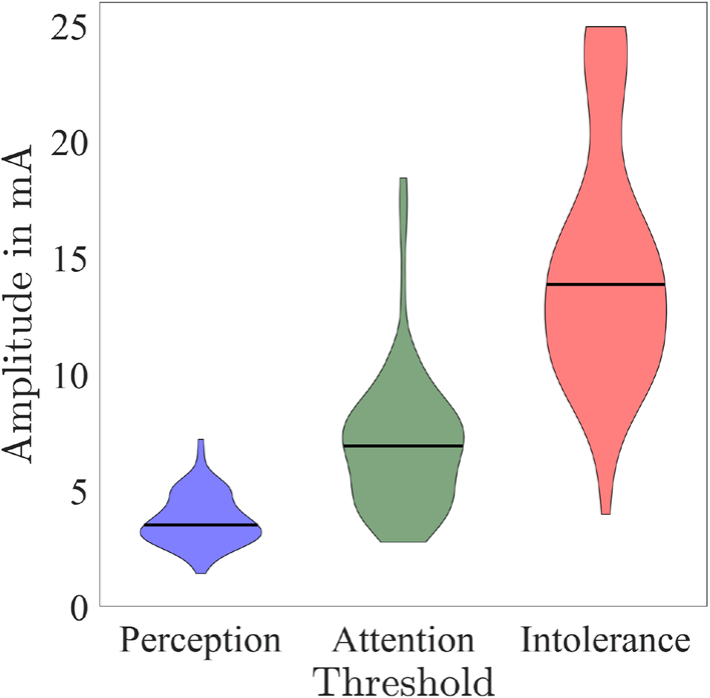
Distribution of perception (blue), attention (green), and intolerance thresholds (red) obtained during the reference threshold determination. The data represent the average over the last 3 measurements in the reference experiment at the beginning of each session, see text.

**Figure 4:**
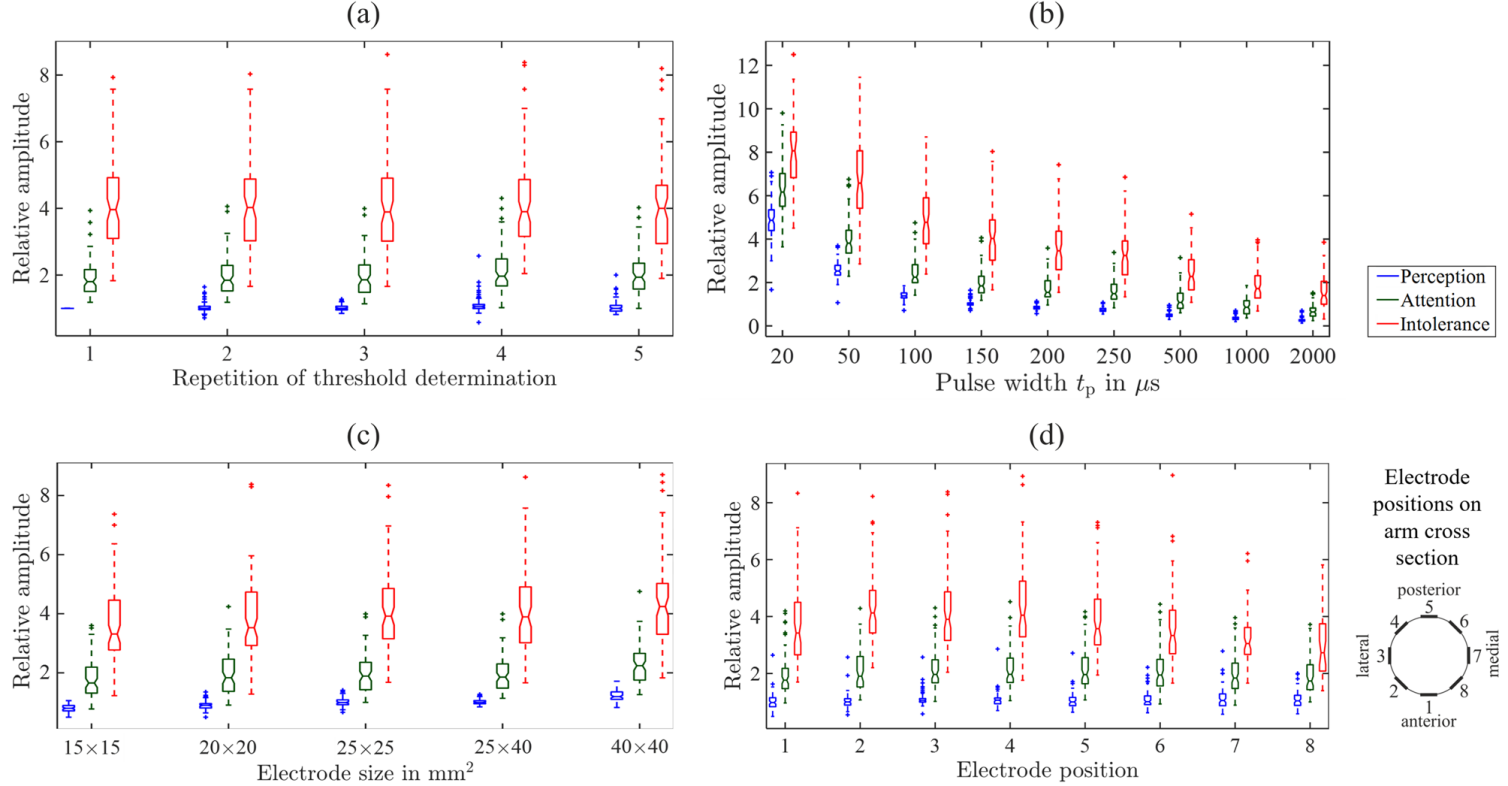
Relative thresholds, normalized by the reference perception threshold as a function of (**a**) repeated threshold determinations during the course of the experiment with electrode pair 3 (25 mm $\times$ 40 mm), (**b**) varying pulse widths, (**c**) varying electrode sizes, and (**d**) electrode position. Please note that the *x*-axis is not to scale for (**b**,**c**).

Figure 3 shows the distribution of the individual mean values of the perception, attention, and intolerance thresholds in mA at electrode pair 3. The median [lower quartile–upper quartile] values of the perception thresholds are 3.5 [2.9–4.5] mA, 6.9 [4.65–8.3] mA, 13.85 [10.85–16.85] mA for perception, attention, and intolerance threshold. During the experiment, the thresholds at electrode pair 3 have been determined five times, see Fig. 4a. Comparing the notches of the Box–Whisker-plots regarding the five repetitions, repetition no. 4 (corresponding to the sub-experiment: Thresholds as a function of the electrode position) shows an increased median value (1.08) of the relative perception threshold compared to the other repetitions (repetition 2: 1.02, repetition 3: 1.02, repetition 5: 1.00). A comparison to repetition no. 1 cannot be drawn for the perception threshold as all thresholds were normalized to these values to calculate the relative threshold. In consequence, the relative perception threshold values of repetition no. 1 are 1 for all participants. The confidence intervals, i.e. the notches of the Box–Whisker-plots, of the median values of the five repetitions of the relative attention thresholds do overlap. This is also the case for the intolerance thresholds. Therefore, no statistical significant difference of the median values of the five repetitions could be found for the attention and the intolerance thresholds.

#### Pulse width

The three relative thresholds, normalized to the reference perception threshold, are shown in Fig. 4 b for varying pulse widths. The relative perception threshold decreased from 4.86 [4.39–5.35] at 20 μs to 0.26 [0.23–0.29] at 2000 μs, whereas the relative attention threshold decreased from 6.17 [5.51–7.02] to 0.64 [0.45–0.83], and the relative intolerance threshold from 8.06 [6.83–8.93] to 1.40 [1.00–2.06]. This decrease is also reflected by the confidence intervals of the median relative thresholds at the corresponding pulse widths, where a ‘=’ indicates that the notches of the Box–Whisker-Plots did overlap (no significant difference). The confidence intervals of the median relative perception thresholds $A_{\text {p}}$ show:

$$A_{\text {p, }20 {\,\upmu \textrm{s}}}>A_{\text {p, }50 {\,\upmu \textrm{s}}}>A_{\text {p, }100 {\,\upmu \textrm{s}}}>A_{\text {p, }150 {\,\upmu \textrm{s}}}>A_{\text {p, }200 {\,\upmu \textrm{s}}}>A_{\text {p, }250 {\,\upmu \textrm{s}}}>A_{\text {p, }500 {\,\upmu \textrm{s}}}>A_{\text {p, }1000 {\,\upmu \textrm{s}}}>A_{\text {p, }2000 {\,\upmu \textrm{s}}}.$$

The confidence intervals of the median relative attention thresholds $A_{\text {a}}$ show:

$$A_{\text {a, }20 {\,\upmu \textrm{s}}}>A_{\text {a, }50 {\,\upmu \textrm{s}}}>A_{\text {a, }100 {\,\upmu \textrm{s}}}>A_{\text {a, }150 {\,\upmu \textrm{s}}}>A_{\text {a, }200 {\,\upmu \textrm{s}}}=A_{\text {a, }250 {\,\upmu \textrm{s}}}>A_{\text {a, }500 {\,\upmu \textrm{s}}}>A_{\text {a, }1000 {\,\upmu \textrm{s}}}>A_{\text {a, }2000 {\,\upmu \textrm{s}}}.$$

The confidence intervals of the median relative intolerance thresholds $A_{\text {i}}$ show:

$$A_{\text {i, }20 {\,\upmu \textrm{s}}}>A_{\text {i, }50 {\,\upmu \textrm{s}}}>A_{\text {i, }100 {\,\upmu \textrm{s}}}>A_{\text {i, }150 {\,\upmu \textrm{s}}}=A_{\text {i, }200 {\,\upmu \textrm{s}}}=A_{\text {i, }250 {\,\upmu \textrm{s}}}>A_{\text {i, }500 {\,\upmu \textrm{s}}}>A_{\text {i, }1000 {\,\upmu \textrm{s}}}=A_{\text {i, }2000 {\,\upmu \textrm{s}}}.$$

Table 2 shows the results of linear regression $y=\beta _0+\beta _1 \cdot x$ with the auxiliary variable for the pulse width $x=1/t_\text {p}$. The explained variance in terms of the coefficient of determination $\hbox {R}^{2}$ decreases from perception to attention and intolerance thresholds with values of 0.94, 0.83, and 0.53. In accordance, the RMSE increases with values of 0.36, 0.75, and 1.58. According to the t-tests of the regression coefficients, the null hypothesis that coefficients are zero could be rejected in all cases. Thus, the fitted non-linear functions and the decrease of the thresholds with increasing pulse width are considered to be significant.

**Table 2:** Statistical analysis of the linear regression $y=\beta _0+\beta _1 \cdot x$ with the auxiliary variable for the pulse width $x=1/t_\text {p}$ calculated for the perception, attention, and intolerance threshold evaluated by RMSE, $\hbox {R}^{2}$, and a t-test of the regression coefficients $\beta _0$ and $\beta _1$ (*p* values).

Threshold	Fitted function	RMSE	$\hbox {R}^{2}$	p-value ($\beta _0$)	*p* value ($\beta _1$)
Perception	$0.37 + 93.20 \cdot 1/t_\text {p}$	0.36	0.94	$\ll 0.0083$	$\ll 0.0083$
Attention	$1.09 + 111.54 \cdot 1/t_\text {p}$	0.75	0.83	$\ll 0.0083$	$\ll 0.0083$
Intolerance	$2.65 + 134.17 \cdot 1/t_\text {p}$	1.58	0.53	$\ll 0.0083$	$\ll 0.0083$

#### Electrode size

The relative thresholds for varying electrode sizes are shown in Fig. 4c. The relative perception threshold increases from 0.80 [0.71–0.89] at 15 mm $\times$ 15 mm to 1.20 [1.10–1.36] at 40 mm $\times$ 40 mm. The confidence intervals of the median values of the relative perception threshold show the following behavior: $A_{\text {p, } 15 \,\text {mm} \times 15 \,\text {mm}}<A_{\text {p, } 20 \,\text {mm} \times 20 \,\text {mm}}<A_{\text {p, } 25 \,\text {mm} \times 25 \,\text {mm}}=A_{\text {p, } 25 \,\text {mm} \times 40 \,\text {mm}}<A_{\text {p, } 40 \,\text {mm} \times 40 \,\text {mm}}$, where a ’=’ indicates that the notches of the Box–Whisker-Plots did overlap (no significant difference). The median values of the relative attention threshold for electrodes of sizes 15 mm $\times$ 15 mm (1.66) and 25 mm $\times$ 40 mm (1.86) are significantly smaller compared to the median value at 40 mm $\times$ 40 mm (2.24). The median values of the relative intolerance threshold for electrodes of sizes 15 mm $\times$ 15 mm (3.31) and 20 mm $\times$ 20 mm (3.53) are significantly smaller compared to the median value at 40 mm $\times$ 40 mm (4.24).

#### Electrode position

The relative thresholds for the different electrode positions are shown in Fig. 4d. For relative perception and attention thresholds no significant difference between the electrode positions can be observed (Fig. 4d, blue and green) as the confidence intervals of the corresponding median values do not overlap. For the relative intolerance threshold, the median values are larger around electrode positions 2, 3, 4, and 5 compared to 1 and 6 to 8 (Fig. 4d, blue).

### Qualitative and spatial perception

#### Reference experiment and repetition of threshold determination

**Figure 5:**
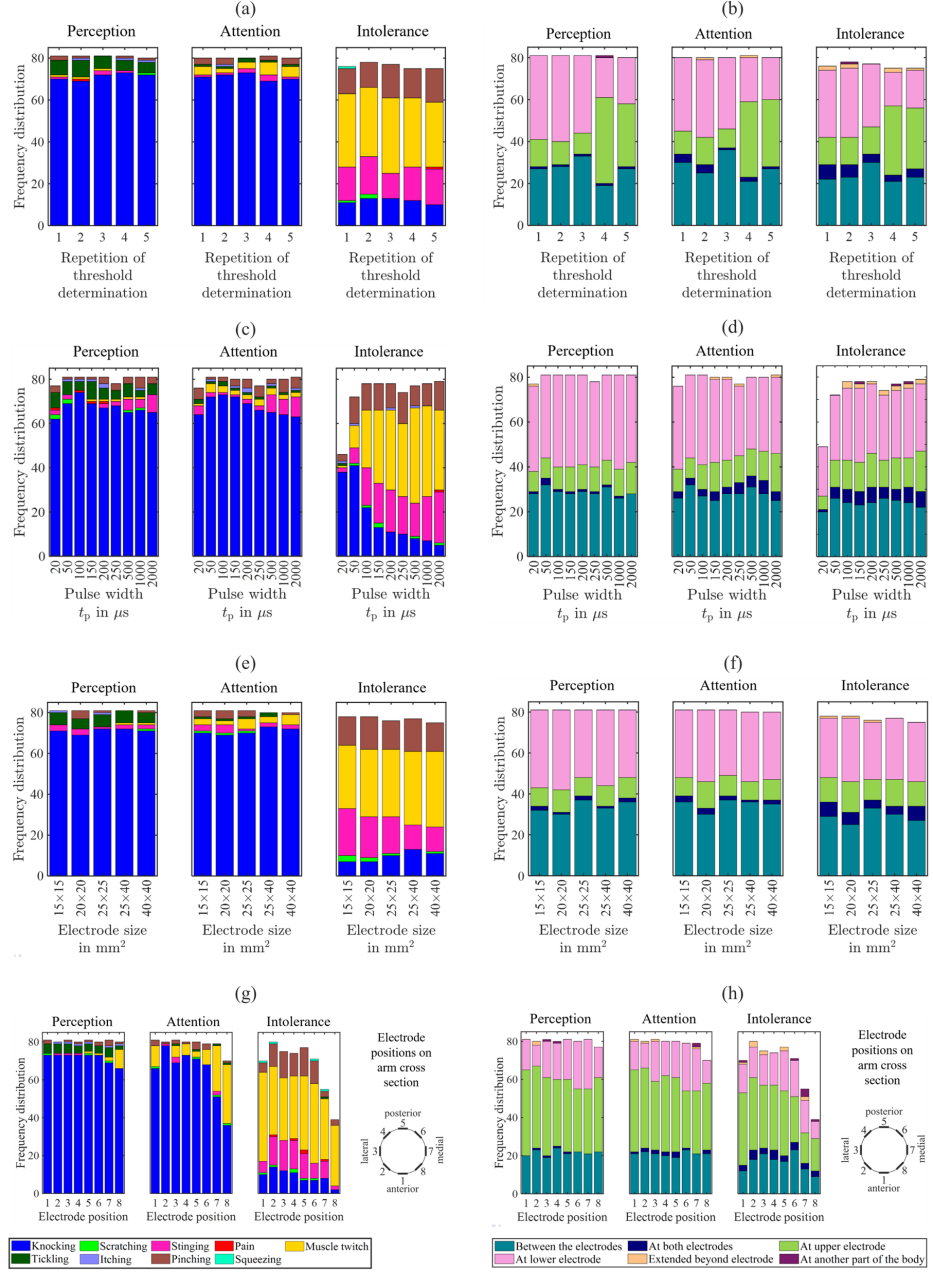
Qualitative (left column) and spatial (right column) perceptions for perception, attention and intolerance thresholds as absolute frequency distributions (upper limit is the number of participants ($n=81$)) as a function of the: (**a**,**b**) repeated threshold determinations during the course of the experiment with electrode pair 3 (25 mm × 40 mm), (**c**,**d**) pulse width, (**e**,**f**) electrode size, and (**g**,**h**) electrode position. Please note that the *x*-axis is not to scale for (**c**–**f**).

The most frequent qualitative perception for perception and attention thresholds is ‘Knocking’ reported by 85–90% of the participants (Fig. 5a, blue) followed by ‘Tickling’ with 6–10% for the perception threshold (Fig. 5a, dark green), and ‘Muscle Twitching’ with 2–7% for the attention threshold (Fig. 5a, yellow). At the intolerance threshold, the dominant perception is ‘Muscle twitching’ for 39–44% followed by ‘Pinching’ 15–20%, and ‘Stinging’ with 15–22%.

Due to technical reasons, the perception threshold was not determined in one case for the repetition 5 (Fig. 5a,b). The missing cases at the intolerance threshold occur due to ‘Muscle twitching’ at the attention threshold, which led to a stop of the measurement series. For the five repetitions, no indication for change of the qualitative perceptions was observed.

The most frequent spatial perception for all three thresholds and repetitions 1–3 (Fig. 5b) is ‘At lower electrode’ for 37–51%, followed by ‘Between the electrodes’ with 27–44%, and by ‘At upper electrode’ with 11–16%. For repetitions 4 and 5, when all 8 electrode pairs were attached, the most frequent spatial perception was ‘At upper electrode’ with 36–51%, followed by ‘Between the electrodes’ with 26–33%, and by ‘At lower electrode’ with 20–26%. The perception ‘At another part of the body’ occurred twice. Two volunteers reported perceptions at the lower electrode of electrode pair 1 and muscle twitching of the thumb.

#### Pulse width

The most frequent qualitative perception for perception and attention threshold is ‘Knocking’ reported by 77–91% of the participants (Fig. 5c, blue), followed by ‘Tickling’ with 4–10% for the perception threshold (Fig. 5c, dark green). For both thresholds, an increase of ‘Stinging’ can be observed starting from $250\,\upmu \hbox {s}$ with 2% to 10–11% at 2000 μs. At the intolerance threshold, the dominant sensation is ‘Muscle twitch’ from 100 μs onwards (Fig. 5c, yellow) for 32–53%. The proportion of ‘Knocking’ decreases at the intolerance threshold from 22% at 100 μs to 6% at 2000  μs. The proportion of ‘Stinging’ from 100  μs onwards lies between 19–28% and of ‘Pinching’ between 11 and 17%.

For pulse width $t_{{\mathrm{p}}}=20\,\upmu \hbox {s}$, the missing cases at the perception and attention threshold (Fig. 5c) indicate where these thresholds were not reached (>25 mA) for 5% and 6% of the participants. Similarly, the missing cases for pulse widths $t_{{\mathrm{p}}}<100\,\upmu \hbox {s}$ at the intolerance threshold (Fig. 5c) indicate that this threshold was not reached ($>25\,\hbox {mA}$) for 43% at $t_{{\mathrm{p}}}=20\,\upmu \hbox {s}$ and for 11% at $t_{{\mathrm{p}}}=50\,\upmu \hbox {s}$. Due to technical reasons, the perception thresholds were not determined in three cases for the pulse width of $250\,\upmu \hbox {s}$ (Fig. 5c,d).

The most frequent spatial perception of all three thresholds with pulse width $\le 100\,\upmu \hbox {s}$ (Fig. 5d) is ‘At lower electrode’ for 37–52%, followed by ‘Between the electrodes’ with 27–40%, and by ‘At upper electrode’ with 12–22%. The perception ‘At another part of the body’ occurred three times at the intolerance threshold (Fig. 5d, dark violet) for the same participant in form of muscle twitching of the thumb.

#### Electrode size

The most frequent qualitative perception of perception and attention threshold is ‘Knocking’ reported by 85–90% of the participants (Fig. 5e, blue), followed by ‘Tickling’ with 6–7% for the perception threshold (Fig. 5e, dark green), and ‘Muscle twitch’ with 2–6% for the attention threshold (Fig. 5e, yellow). At the intolerance threshold, the dominant perception is ‘Muscle twitch’ (Fig. 5e, yellow) for 38–46%. The proportion of ‘Stinging’ at the intolerance threshold decreases from 28% at 15 mm × 15 mm to 15% at 40 mm × 40 mm. The proportion of ‘Pinching’ lies between 17 and 20%.

The most frequent spatial perception for all three thresholds (Fig. 5f) is ‘At lower electrode’ for 35–48%, followed by ‘Between the electrodes’ with 31–46%, and ‘At upper electrode’ with 11–19%.

#### Electrode position

The most frequent qualitative perception at the perception and attention thresholds is ‘Knocking’ reported by 44–91% of the participants (Fig. 5g, blue), followed by ‘Tickling’ with 2–6% for the perception threshold (Fig. 5g, dark green), and ‘Muscle twitch’ with 0–38% for the attention threshold (Fig. 5g, yellow). An increase of the proportion of ‘Muscle twitch’ at medial electrode positions can be observed at positions 7 and 8 for the perception threshold and at 1 and 6-8 for the attention threshold. At the intolerance threshold, the dominant perception is ‘Muscle twitch’ (Fig. 5g, yellow) for 40–58% followed by ‘Stinging’ with 2–20%, and ‘Pinching’ with 4–19%. For one female participant with an arm circumference of 20 cm it was not possible to attach all 8 electrode pairs without overlapping. Thus, only electrode pairs 1, 3, 5, and 7 have been placed. In consequence, the thresholds at the positions 2, 4, 6, and 8 are missing (Fig. 5g,h). In three cases muscle twitch could be observed at the perception threshold at electrode position 8. No specific spatial location at the upper arm could be reported by those participants (Fig. 5g,h) because the slight sensation was very indistinct. The missing cases at the attention and intolerance threshold occur due to ‘Muscle twitch’ at the perception and attention threshold, which led to a stop of the current measurement series.

The most frequent spatial perception for all three thresholds (Fig. 5h) is ‘At lower electrode’ for 20–56%, followed by ‘Between the electrodes’ with 11–30%, and by ‘At upper electrode’ with 11–32%. The perception ‘At another part of the body’ occurred twice at the perception threshold, twice at the attention threshold, and 7 times at the intolerance threshold (Fig. 5h, dark violet). At the perception threshold, both cases occurred for the same participant. At electrode pair 3, the spatial perception occurs at the lower electrode of pair 1 and at pair 4 the perception occurs at the upper electrode of pair 1. At the attention threshold, one participant sensed the stimulation at electrode pair 7 at the lower arm, the other participant could not exactly locate the perception. At the intolerance threshold, the reported spatial perceptions at another part of the body were: at the lower arm (2×), muscle twitch near the elbow, at the wrist, muscle twitch of the middle finger, and ring finger, at the fingers (2×).

## Discussion

To derive practical parameter sets for an electrical warning system, we determined the perception, attention, and intolerance thresholds for variable stimulation pulse widths, electrode sizes, and electrode positions. In the field of electrocutaneous stimulation, the perception threshold is widely known, often reported as sensory threshold^16,17^. Further thresholds used in other studies are the motion and pain threshold as reported in^16,17^. The novel attention and intolerance thresholds were specifically defined for the development of a wearable warning system and have been introduced in our previous pilot study^6^.

We used symmetric, biphasic rectangular pulses since they have the lowest total charge among the five commonly used waveforms^18,19^. Charge-balanced biphasic pulses are important to avoid tissue injury or electrode damage. In future research, alternative biphasic waveforms, like shown in^20^ might be considered to decrease charge loss outside of the tissue of interest and, therefore, reduce material fatigue and support biocompatibility for long-term use.

We aimed at a local perception effect at the chosen location at the upper arm with our electrocutaneous stimulation. Previous literature^21–24^ indicates that bipolar stimulation is well suited to obtain a local perception effect. Consequently, we used a bipolar arrangement of the electrode pairs (here: distance of 1 cm). Our results confirm the expected local perception effect (cf. Fig. 5a,c,e,g).

The repetition of the threshold determination (cf. Fig. 4a) at electrode pair 3 showed an increased median relative perception threshold at repetition no. 4. The difference at this threshold determination is that thresholds have been determined consecutively at electrode pairs 1 to 8 whereas for repetitions 1, 2, 3, and 5 the thresholds were only determined at pair 3 while other parameters were changed. This might be a hint that the stimulation of close electrode pairs before could alter the perception thresholds at the electrode of interest. Given the fact that the future warning system is assumed to work in the range between attention and intolerance thresholds, this change of perception threshold is considered minor.

The investigated pulse widths from 20 to $2000\,\upmu \hbox {s}$ were chosen to be in the range of other applications of electrocutaneous stimulation^11,21,25,26^. The thresholds showed a non-linear decrease with increasing pulse width (Fig. 4b), which was also observed in^27–29^. The decrease was statistically quantified by a linear regression $y = \beta _0 +\beta _1 \cdot x$, where the pulse width $t_\text {p}$ was replaced by the auxiliary variable $x = 1/t_\text {p}$. The values of RMSE increase from perception to attention and intolerance thresholds. In accordance, the $\hbox {R}^{2}$, which reflects the explained variability decreased from 0.94 to 0.53. This result mirrors the increasing variance of the study from perception to attention and intolerance thresholds. Another relation was shown in^5^ where the perception threshold decreased with an increasing number of pulses. Both relations reflect the assumption^30^ that a similar charge quantity is necessary to perceive an electrical stimulation of similar intensity. In contrast^29^, assumes that the product $I^2 \cdot t_{{\mathrm{p}}}$ needs to remain constant to perceive an electrical stimulation of similar intensity, where *I* denotes the current amplitude and $t_{{\mathrm{p}}}$ the pulse width. The decreasing slope gets smaller for larger pulse widths (Fig. 4b). This might be explained partly by the capacitive properties of the boundary layer between electrode and skin, which leads to spatially broader, distributed, and, thus, locally weaker charge distributions^31^.

The difference between the attention and intolerance threshold can be seen as a range of operation for the electric warning system and is smaller for higher pulse widths. Therefore, they are less suitable for a warning system. On the other end, very short pulse widths are also less suitable because they require higher current amplitudes, sometimes exceeding the planned upper limit of 25 mA. Therefore, pulse widths of about $t_{{\mathrm{p}}}= 150\,\upmu \hbox {s}$ to $250\,\upmu \hbox {s}$ appear suitable and we have chosen $t_{{\mathrm{p}}}= 150\,\upmu \hbox {s}$ as a standard value for further experiments.

The perception threshold shows a slight increase with increasing electrode size. This is in agreement with results from electrocutaneous stimulation of the sole of the foot^32^. For the attention threshold, the median values were larger for electrodes of size 40 mm × 40 mm compared to 15 mm × 15 mm and 25 mm × 40 mm. For the intolerance threshold the median values were larger for electrodes of size 40 mm × 40 mm compared to 15 mm $\times$ 15 mm and 20 mm × 20 mm. However, given the fact that the Box–Whisker-plots for the attention and intolerance overlap at all electrode sizes, we conclude that all of the investigated electrode sizes are usable. The future warning system is assumed to work in the range between attention and intolerance thresholds, thus electrode sizes between 15 mm × 15 mm and 40 mm × 40 mm can be considered.

The electrode position showed no influence on perception and attention threshold, which is in contrast to electrocutaneous stimulation of the forearm reported in Geng et al. 2011^5^. The intolerance threshold was increased at lateral electrode positions 2, 3, 4, and 5 compared to the medial positions 1 and 6-8. However, given that we stopped the measurement series as soon as visible muscle twitching occurred, the actual intolerance threshold was not reached in all cases. The corresponding participants reported that their intolerance threshold is not yet reached, and the slightly visible muscle twitching does not feel uncomfortable. Consequently, future studies should consider slight muscle twitches to be acceptable for an electric warning system.

Across all experiments, the attention and intolerance threshold distributions partly overlap, reflected especially in the Whisker parts of the boxplots (Fig. 4). This means that a future wearable solution cannot use a single working range of stimulation amplitudes for all users. The parameters need to be adjusted individually.

Across our experiments, the most frequent qualitative perception of perception and attention threshold was ‘Knocking’ (44–91%). This result is comparable to the finding in^11^, where ‘Touch’ was reported as one of the most frequent qualitative perceptions. At the intolerance threshold, ‘Muscle twitch’ (32–58%), ‘Stinging’, and ‘Pinching’ (15–28%) appeared as the most frequent qualitative perceptions. This stability of the most qualitative perceptions is beneficial for the further development of an electrical warning system, as it allows us to easily instruct novel users. This stability was also present for the reference threshold determination at the beginning of the experiment, where the mean values of the last three stable thresholds were determined for the perception, attention, and intolerance thresholds and the most frequent qualitative perception out of three was selected. For the case that three varying qualitative perceptions occurred, the last one was selected. This case did not happen for the perception and the attention threshold and only in 1.2% for the intolerance thresholds.

Muscle twitches could not be avoided completely in our study. They also appeared before reaching the attention or the intolerance threshold (Fig. 5). As the working range of the future warning system is expected to be between the individual attention and intolerance threshold, we conclude that the current configuration investigated in this study is not suitable for warning workers during fine motor tasks. Therefore, the focus of the further development shifts to the warning of workers during gross sensory-motor tasks. It should be investigated if minor muscle twitches represent a problem for the workers during their occupational activities. In consequence, the association between the three thresholds and the motor threshold should be separated in future studies. Muscle twitches appeared most frequently at medial positions 1 and 5–8 (Fig. 5g) at all thresholds. Thus, if muscle twitches need to be avoided, the stimulation electrodes should be preferably placed at the lateral electrode positions 2, 3, and 4.

For the spatial perceptions, an increase of the perception frequency of ‘At the upper electrode’, accompanied by a decrease of ‘Between the electrodes’ and ‘At the lower electrode’ was observed for the repetition of the threshold determination (Fig. 5b). This might be explained partly by the fact that before repetition step 3, all 8 electrode pairs were attached to the upper arm, which might influence the spatial perception. However, most spatial perceptions were reported at ‘At the lower electrode’, ‘At the upper electrode’, or ‘Between the electrodes’. Only in 0.7% of all electrocutaneous stimulations, a perception at another part of the body was reported. For the reference threshold determination at the beginning of the experiment, the mean values of the last three stable thresholds were determined for the perception, attention, and intolerance thresholds and the most frequent spatial perception out of three was selected. For the case that three varying spatial perceptions occurred, the last one was selected. This case did not happen for the perception threshold, in 6.2% of the attention thresholds and in 1.2% of the intolerance thresholds. This indicates that the spatial perception of the stimulus is consistent, which is of importance for the repeatable application of a future warning system. Spatial perception depends on the individual anatomy of the participants, thus, how receptors, fat, muscles, and nerves are distributed below the electrodes. However, most of the stimulations lead to perceptions close to the electrode location. This is in accordance with the results reported in^11^. The participants gave feedback that it was difficult to report the spatial perception accurately. We conclude that a spatial coding of a warning signal in the scale of one electrode pair may not be reliable, which, however, is not a problem for a future warning system.

There are some limitations to our study. The investigated electrode configurations were limited to vertical pairs and only a selection of electrode sizes which was based on practical considerations. Other configurations like transversal or diagonal electrode pairs or the influence of the neighboring margin lengths of the electrodes should be investigated in future studies. Further, the experiments have been conducted at the lateral electrode pair no. 3 (except for the electrode position investigation) with a pulse width of $150\,\upmu \hbox {s}$ (except for the pulse width investigation). Although future studies need to investigate the effects of pulse width for all electrode positions, we believe that a pulse width of $150\,\upmu \hbox {s}$ can be chosen as a basic working parameter for a future warning system. Factors like age dependency were not investigated in this study because 79% of the study group are 20–30 years of age. Future studies will try to provide a more uniform distribution of participants between 18 and 65 years of age as other studies of electrocutaneous stimulation showed age-related effects of the perception threshold^33,34^. Regarding gender, no indications could be found for differences between the thresholds in this study. However, the gender groups were not equally distributed (female: 29, male: 52). More research is needed because other studies showed gender differences^17,34,35^.

The results of this study might be also beneficial for other fields of electrocutaneous stimulation, such as tactile feedback in prosthetics, or nerve and muscle stimulation.

## Conclusions

This study showed the realizability of an electric warning system and found suitable parameters of electrocutaneous stimulation of the upper arm with a biphasic current stimulation pulse during rest. Future studies will focus on the transferability of the results to textile cuff electrodes and investigate the question how minor muscle twitches influence the realizability of the stimulation in a working situation.

## Supplementary Information

Supplementary Figure.
