# Publisher Correction: Perception thresholds and qualitative perceptions for electrocutaneous stimulation

**DOI:** 10.1038/s41598-022-14650-8

**Published:** 2022-06-15

**Authors:** Eva‑Maria Dölker, Stephan Lau, Maria Anne Bernhard, Jens Haueisen

**Affiliations:** 1 Institute of Biomedical Engineering and Informatics, Technische Universität Ilmenau, 98693 Ilmenau, Germany; 2 Australian Institute for Machine Learning (AIML), School of Computer Science, The University of Adelaide, Adelaide, 5005 Australia

Correction to: *Scientific Reports* 10.1038/s41598-022-10708-9, published online 05 May 2022

The original PDF version of this Article contained errors in the Results section under the subheading ‘Pulse width’, where the confidence intervals of the median relative perception thresholds ${A}_{\text{p}}$ are illegible. These now read:

$$A_{{{\text{p}},\,20\,\upmu {\text{s}}}} > A_{{{\text{p}},\,50\,\upmu {\text{s}}}} > A_{{{\text{p}},\,100\,\upmu {\text{s}}}} > A_{{{\text{p}},\,150\,\upmu {\text{s}}}} > A_{{{\text{p}},\,200\,\upmu {\text{s}}}} > A_{{{\text{p}},\,250\,\upmu {\text{s}}}} > A_{{{\text{p}},\,500\,\upmu {\text{s}}}} > A_{{{\text{p}},\,1000\,\upmu {\text{s}}}} > A_{{{\text{p}},\,2000\,\upmu {\text{s}}}}$$.

The confidence intervals of the median relative attention thresholds ${A}_{\text{a}}$ show:

$$A_{{{\text{a}},\,20\,\upmu {\text{s}}}} > A_{{{\text{a}},\,50\,\upmu {\text{s}}}} > A_{{{\text{a}},\,100\,\upmu {\text{s}}}} > A_{{{\text{a}},\,150\,\upmu {\text{s}}}} > A_{{{\text{a}},\,200\,\upmu {\text{s}}}} = A_{{{\text{a}},\,250\,\upmu {\text{s}}}} > A_{{{\text{a}},\,500\,\upmu {\text{s}}}} > A_{{{\text{a}},\,1000\,\upmu {\text{s}}}} > A_{{{\text{a}},\,2000\,\upmu {\text{s}}}}$$.

The confidence intervals of the median relative intolerance thresholds ${A}_{\text{i}}$ show:

$$A_{{{\text{i}},\,20\,\upmu {\text{s}}}} > A_{{{\text{i}},\,50\,\upmu {\text{s}}}} > A_{{{\text{i}},\,100\,\upmu {\text{s}}}} > A_{{{\text{i}},\,150\,\upmu {\text{s}}}} = A_{{{\text{i}},\,200\,\upmu {\text{s}}}} = A_{{{\text{i}},\,250\,\upmu {\text{s}}}} > A_{{{\text{i}},\,500\,\upmu {\text{s}}}} > A_{{{\text{i}},\,1000\,\upmu {\text{s}}}} = A_{{{\text{i}},\,2000\,\upmu {\text{s}}}}$$.

Table 2 shows the results of linear regression $y={\beta }_{0}+{\beta }_{1}\cdot x$ with the auxiliary variable for the pulse width $x=1/{t}_{\text{p}}$.

The original PDF version of this Article has been corrected.

