# Supplementary figures and images for: Perception thresholds and qualitative perceptions for electrocutaneous stimulation
Source: Sci Rep. 2022 May 5;12:7335. doi: 10.1038/s41598-022-10708-9 (PMC9072403; doi:10.1038/s41598-022-10708-9)

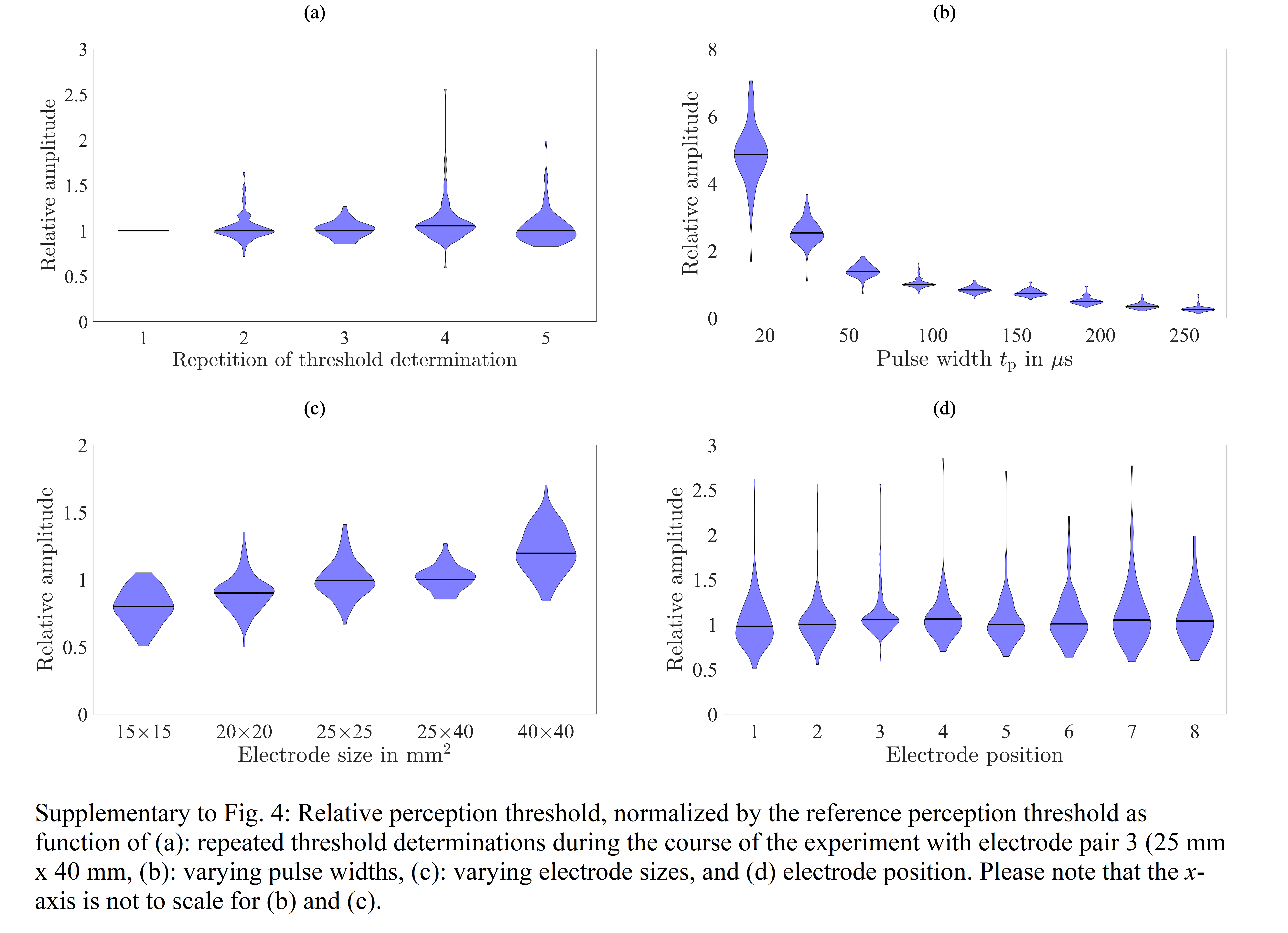

Supplement: Supplementary file 1 — Supplementary Figure. [file 41598_2022_10708_MOESM1_ESM.png]
